# Post-transcatheter aortic valve implantation isolated PR prolongation: incidence and clinical significance

**DOI:** 10.1093/europace/euae011

**Published:** 2024-01-16

**Authors:** Nimrod Perel, Oholi Tovia-Brodie, Asher Schnur, Moshe Rav Acha, Nir Levi, Yogev Cohen, Danny Dvir, Michael Glikson, Yoav Michowitz

**Affiliations:** Jesselson Integrated Heart Center, Shaare Zedek Medical Center, 12 Shmuel Beit Street, Jerusalem, Israel; Faculty of Medicine, Hebrew University, Jerusalem, Israel; Jesselson Integrated Heart Center, Shaare Zedek Medical Center, 12 Shmuel Beit Street, Jerusalem, Israel; Faculty of Medicine, Hebrew University, Jerusalem, Israel; Department of Internal Medicine C, Shaare Zedek Medical Center,12 Shmuel Beit Street, Jerusalem, Israel; Faculty of Medicine, Hebrew University, Jerusalem, Israel; Jesselson Integrated Heart Center, Shaare Zedek Medical Center, 12 Shmuel Beit Street, Jerusalem, Israel; Faculty of Medicine, Hebrew University, Jerusalem, Israel; Jesselson Integrated Heart Center, Shaare Zedek Medical Center, 12 Shmuel Beit Street, Jerusalem, Israel; Faculty of Medicine, Hebrew University, Jerusalem, Israel; Faculty of Medicine, Hebrew University, Jerusalem, Israel; Jesselson Integrated Heart Center, Shaare Zedek Medical Center, 12 Shmuel Beit Street, Jerusalem, Israel; Faculty of Medicine, Hebrew University, Jerusalem, Israel; Jesselson Integrated Heart Center, Shaare Zedek Medical Center, 12 Shmuel Beit Street, Jerusalem, Israel; Faculty of Medicine, Hebrew University, Jerusalem, Israel; Jesselson Integrated Heart Center, Shaare Zedek Medical Center, 12 Shmuel Beit Street, Jerusalem, Israel; Faculty of Medicine, Hebrew University, Jerusalem, Israel

**Keywords:** Transcatheter aortic valve implantation, PR interval, Conduction abnormality, Pacemaker

## Abstract

**Aims:**

Conduction abnormalities post-transcatheter aortic valve implantation (TAVI) are common. Post-TAVI PR prolongation was mainly studied as an adjunct to new-onset bundle branch block. The net effect of isolated PR prolongation (IPRP) without post-TAVI QRS changes is not well known. The aim of this study was to define the incidence and clinical significance of post-TAVI IPRP.

**Methods and results:**

A total of 1108 consecutive TAVI patients were reviewed. Patients with IPRP were compared with patients without post-TAVI electrocardiogram (ECG) changes. Clinical outcomes included permanent pacemaker implantation (PPI) and overall mortality. A total of 146 patients with IPRP were compared with 290 patients without post-TAVI ECG changes. At 1 year follow-up, 4 (2.7%) and 7 (2.4%) patients underwent PPI (*P* = 0.838) and 10 (6.8%) and 25 (8.6%) died (*P* = 0.521), from the study and control groups, respectively. No patient with IPRP and narrow QRS underwent PPI during 1 year post-TAVI, and all death events were non-cardiac except one unknown cause. Permanent pacemaker implantation rates among patients with IPRP and wide QRS were higher (*n* = 4, 12.1%), compared with patients with wide QRS without post-TAVI ECG change (*n* = 3, 4%) however not reaching statistical significance (*P* = 0.126). Multivariate Cox proportional hazards model demonstrated that in patients with narrow QRS, neither PR prolongation nor baseline or maximal PR intervals was associated with the combined endpoint of PPI and mortality. However, in patients with wide QRS, baseline PR intervals and QRS width, but not PR prolongation were associated with the combined outcome.

**Conclusion:**

Post-TAVI IPRP in patients with narrow QRS is not associated with adverse outcome. This finding may translate clinically into a more permissive approach to these patients.

## Graphical Abstract

**Graphical Abstract:**
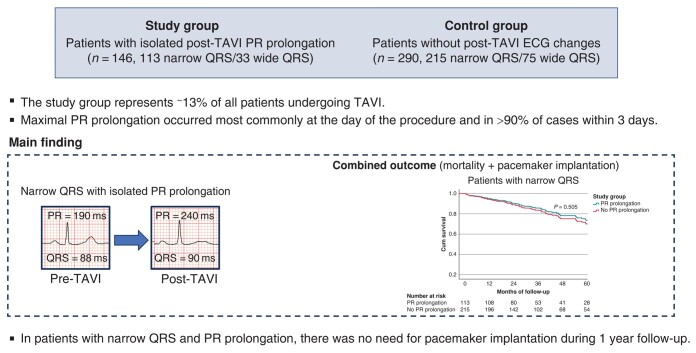

**What’s new?**

- Post-transcatheter aortic valve implantation (TAVI) PR prolongation was mainly studied as an adjunct to new-onset bundle branch block. The clinical significance of post-TAVI isolated PR prolongation without a change in QRS is unknown.
- Isolated PR prolongation of 40.2 ± 22 ms may occur in ∼13% of patients undergoing TAVI, with maximal prolongation occurring most commonly at the day of the procedure and in >90% of cases within 3 days.
- Among patients with narrow QRS, isolated PR prolongation and baseline or maximal PR intervals were not found to be associated with increased risk for pacemaker implantation or mortality.
- In patients with wide QRS, baseline PR interval but not PR prolongation was associated with increased risk for mortality or pacemaker implantation.

## Introduction

Conduction abnormalities (CA) after transcatheter aortic valve implantation (TAVI) are common, occurring in 10–40% of patients.^1–6^ New-onset left bundle branch block (LBBB) is the most studied CA, and several reports suggested that the magnitude of QRS duration and/or accompanied PR prolongation increase the risk of complete heart block (CHB), permanent pacemaker implantation (PPI), and mortality.^1,2,7–9^

PR prolongation as a risk factor for the development of post-TAVI CHB was mainly studied as an adjunct to new-onset LBBB or baseline right bundle branch block (RBBB), demonstrating higher atrio-ventricular block (AVB) risk in both situations.^7,10–12^ The few studies reporting on patients with prolonged PR and narrow QRS included only a limited patient cohort, and it was unknown whether there were any post-procedural dynamic QRS duration changes that fell below 120 ms (i.e. a patient with PR > 200 ms and dynamic changes in QRS from baseline of 80 to110 ms or axis change would fit into this subgroup).^7,10^

The latest European guidelines on cardiac pacing^13^ recommend that patients with post-TAVI LBBB and QRS and/or PR intervals longer than 150 and 240 ms, respectively, undergo electrophysiological study (EPS) or prolonged monitoring. Likewise, a IIb recommendation exists for patients with baseline CA who prolong their PR or QRS by more than 20 ms. However, no recommendation on the approach to patients with isolated PR prolongation without baseline CA exists in the guidelines and its significance is currently unknown.

The aim of the current study was to evaluate the incidence and timing of post-TAVI isolated PR prolongation and its association with adverse outcome.

## Methods

### Study population

All patients who underwent TAVI between the years 2010 and 2021 in our medical centre (Shaare Zedek Medical Center, Jerusalem) were candidates for inclusion. The study protocol was approved by the local ethics committee, and the need for written informed consent was waived as the study was a retrospective analysis of deidentified data. Clinical, laboratory, and echocardiographic data were collected from patients’ medical records.

Exclusion criteria included age < 18 years, pre-TAVI PPI, evidence for atrial fibrillation (AF) on pre- or post-TAVI electrocardiogram (ECG) (precluding assessment of PR interval), patients who had post-TAVI QRS widening of more than 20 ms (see below), no ECG or ECG of low quality in patients’ records, and patients who died during the procedure or developed immediate post-TAVI AVB and had paced rhythm on their first post-TAVI ECG.

### Electrocardiogram analysis

All patients had routine ECG performed within a week before the TAVI procedure (baseline ECG), immediately post-TAVI upon admission to the intensive care unit or cardiology department, and daily thereafter until hospital discharge. All ECGs were stored digitally in patients’ files.

Compared with baseline ECG, post-TAVI ECGs were evaluated for PR and/or QRS prolongation defined as at least 20 ms prolongation, on any of the pre-discharge post-TAVI ECGs. Patients with QRS interval prolongation were excluded. The baseline ECG was used for reporting QRS width and morphology as well as baseline PR interval. In addition, in patients who had isolated PR prolongation, maximal PR interval was reported based on the pre-discharge ECG demonstrating the longest PR interval. Heart rate at baseline ECG and ECG with maximal PR prolongation was compared, and a difference ≥ 20 b.p.m. was reported. In addition, for further defining possible mechanisms of PR prolongation, in patients with isolated PR prolongation who had multiple ECGs performed before the TAVI procedure (≥2 ECG during the 1 year period preceding the procedure besides the baseline ECG), we evaluated whether there was evidence for changes in PR interval (of more than 20 ms) before the procedure.

Further defining QRS morphology into left anterior or posterior haemiblocks, intraventricular conduction delay (IVCD) and left or right BBB was based on the American Heart Association/American College of Cardiology Foundation/Heart Rhythm Society recommendations for the standardization and interpretation of the ECG^14^ and the 2021 European Society of Cardiology (ESC) guidelines on cardiac pacing and cardiac resynchronization therapy.^13^

### Follow-up

Patients were followed for the occurrence of PPI and mortality within 1 year after the procedure as well as for the combined endpoint of total mortality and PPI during follow-up. Follow-up was conducted using chart review or phone call. End of follow-up was marked as the last day on which the patient was seen in any of the hospital clinics or departments as well as health maintenance organization (HMO) clinics or the last phone contact (whichever occurred later). In addition, mortality data were also retrieved from governmental population registry.

### Statistical analysis

Characteristics were described by percentages, means ± SD, and medians with interquartile ranges (IQR). Categorical variables were compared using χ^2^ test or Fisher exact test and continuous variables using unpaired Student’s *t*-test or Mann–Whitney test. Multivariate Cox proportional hazards model was used to assess the effect of isolated PR prolongation on mortality or need for PPI. Factors included in the model were study group, age, sex, diabetes mellitus (DM), hyperlipidaemia, ischaemic heart disease (IHD), hypertension (HTN), QRS duration, baseline or maximal PR duration, ejection fraction (EF), and presence of RBBB morphology. The model was adjusted for potential confounders that were introduced to the model using backward stepwise method. Hazard ratios (HR) with 95% confidence intervals (CIs) were reported. Adjusted survival curves were displayed. Subgroup analyses were performed by applying all comparisons separately on patients with baseline narrow (<120 ms) and wide QRS interval (≥120 ms). The statistical tests were two-sided and *P* < 0.05 was considered statistically significant. Analyses were carried out using SPSS Statistics for Windows, version 29.0 (IBM Corp, Armonk, NY, USA).

## Results

### Study cohort

**Figure 1:**
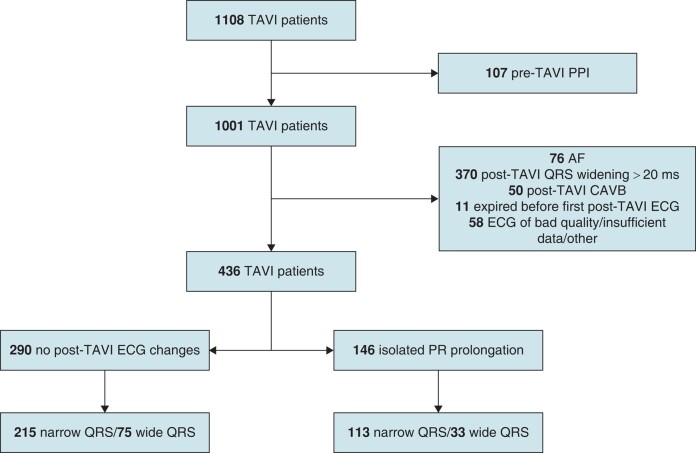
Flowchart demonstrating reasons for exclusion and cohort selection.

**Figure 2:**
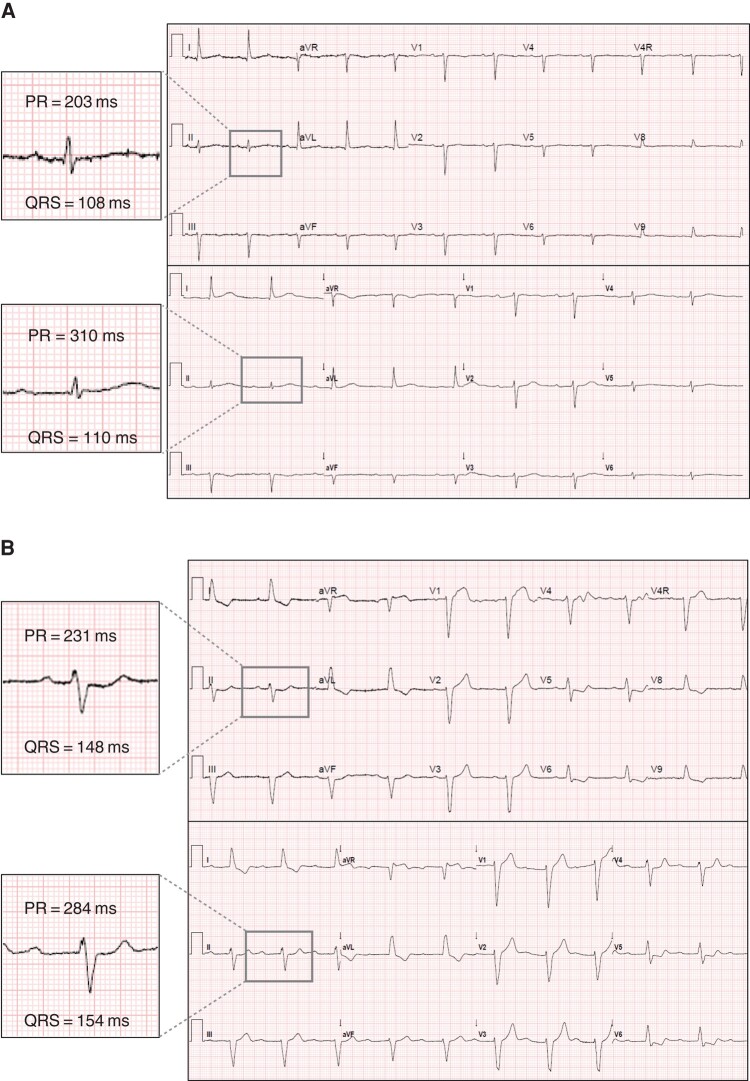

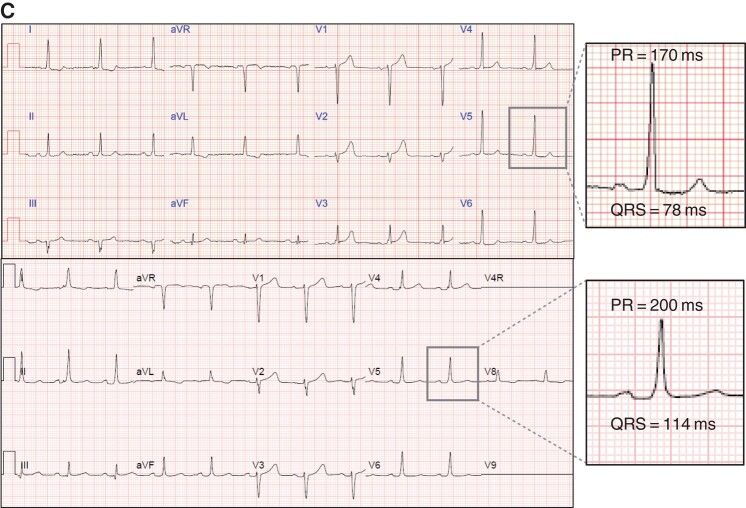
Representative ECG examples of (*A*) patients with narrow QRS and post-TAVI isolated PR prolongation, (*B*) patients with wide QRS of LBBB morphology and post-TAVI isolated PR prolongation, and (*C*) patients with baseline narrow QRS with post-TAVI PR prolongation as well as QRS widening >20 but <120 ms. This patient also had changes in QRS morphology as seen in leads III, aVL, and aVF. As described in the texts, patients with these ECG changes were excluded.

During the study period, 1108 patients underwent TAVI (*Figure 1*). Of whom, 107 had pre-TAVI PPI, 76 were in AF, 50 developed immediate post-TAVI complete AVB (CAVB), 11 expired in the catheterization laboratory or in the operating room, and 54 had ECG of low quality or insufficient information on their digital records. In three, the procedure was aborted and one transapical procedure was also excluded. In addition, 370 patients had QRS interval widening of more than 20 ms, of whom in 14 (3.8%) the QRS widened to <120 ms (*Figure 2*).

Thus, 436 patients were studied (39.4% of the TAVI population), of which 146 belonged to the study group that demonstrated isolated PR prolongation (being 13.2% of the whole TAVI population evaluated in this study) and 290 patients comprised the control group that did not have post-TAVI change of their baseline ECG (*Table 1*). Most patients from the study and control groups had baseline narrow QRS (<120 ms), 77.4% and 74.1%, respectively. Median follow-up was 979 (680.5–1831) and 1010.5 (587.75–1929.25) days, *P* = 0.61, for the study and control groups, respectively. All patient who did not expire had a least 1 year of follow-up except for one and five patients who were lost to follow-up before 1 year post-TAVI (at Days 191 and 242, 296, 22, 35, and 257 post-TAVI, for the study and control group patients, respectively).

**Table 1:** Baseline characteristics of TAVI patients with and without isolated PR prolongation

	Isolated PR prolongation	No PR prolongation	*P* value
	*n* = 146	*n* = 290	
Age	79.2 ± 9	80.8 ± 8	0.09
Sex, female	69 (47.3)	141 (48.6)	0.788
Hyperlipidaemia	85 (58.2)	149 (51.4)	0.176
DM	66 (45.2)	97 (33.4)	**0.017**
HTN	123 (84.2)	229 (79)	0.187
IHD	58 (39.7)	161 (55.5)	**0**.**002**
Echocardiography			
LVEF^a^			
LVEF ≥ 50%	123 (84.2)	220 (75.9)	**0**.**044**
AVA	0.65 ± 0.16	0.6 ± 0.15	**0**.**002**
Peak gradient	80.5 ± 24.7	79.1 ± 24.9	0.569
Mean gradient	47.4 ± 16.6	45.9 ± 15.7	0.599
** **Medications			
Beta-blockers	57 (39)	130 (44.8)	0.249
Ca blockers	2 (1.4)	8 (2.8)	0.361
Amiodarone	7 (4.8)	24 (8.3)	0.182
ECG			
PR interval baseline	179.2 ± 32.2	188 ± 36.9	**0**.**029**
PR interval > 200 ms^b^	94 (64.4)	83 (28.6)	**<0**.**001**
PR interval ≥ 240 ms^b^	37 (25.3)	31 (10.7)	**<0**.**001**
Maximal PR	219.4 ± 40,1	188 ± 36.9	**<0**.**001**
Heart rate baseline	69.3 ± 11.9	71.4 ± 12.7	0.111
Baseline QRS interval	102.4 ± 24.2	103.4 ± 25.6	0.809
QRS interval ≥ 120 ms	33 (22.6)	75 (25.9)	0.457
QRS interval ≥ 150 ms	15 (10.3)	19 (6.6)	0.171
RBBB	16 (48.5)	34 (45.3)	0.282
LBBB	12 (36.4)	36 (48)	
IVCD	5 (15.2)	5 (6.7)	
Valve type			
Sapien/Sapien XT/Sapien 3	71 (48.6)	151 (52.1)	0.383
CoreValve/Evolut R/Evolut Pro/Evolut Pro Plus	44 (30.1)	67 (23.1)	
Portico/Navitor	0 (0)	1 (0.3)	
Acurate Neo I/Acurate Neo II	31 (21.2)	71 (24.5)	
Hospitalization duration	4 (3–6)	4 (3–6)	0.567
Femoral approach	144 (98.6)	272 (93.8)	**0**.**023**
In-hospital PPI	2 (1.4)	4 (1.4)	0.994
1 year PPI	4 (2.7)	7 (2.4)	0.838
Overall PPI	6 (4.1)	12 (4.1)	0.989
1 year mortality	10 (6.8)	25 (8.6)	0.521
Overall mortality	39 (26.7)	87 (30)	0.475
1 year mortality or PPI	13 (8.9)	31 (10.7)	0.559
FU time, days	979 (680.5–1831)	1010.5 (587.75–1929.25)	0.61

AVA, aortic valve area; DM, diabetes mellitus; FU, follow-up; HTN, hypertension; IHD, ischaemic heart disease; IVCD, intraventricular conduction disturbance; LBBB, left bundle branch block; LVEF, left ventricular ejection fraction; PPI, permanent pacemaker implantation; RBBB, right bundle branch block; TAVI, transcatheter aortic valve implantation.

The bold means that it is statistically significant.

^a^Full distribution is presented in Supplementary material online, *Table S1*.

^b^Any PR either at baseline or maximal PR.

### PR prolongation

**Figure 3:**
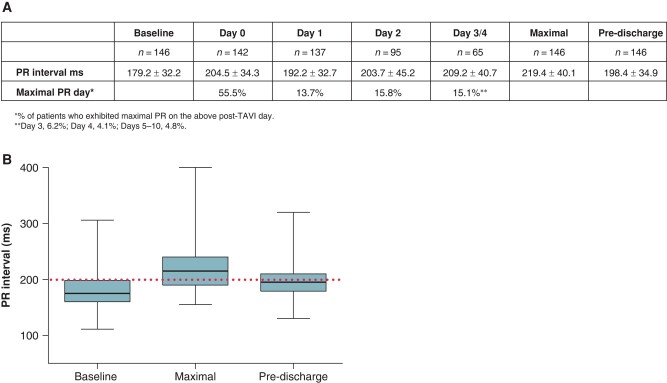
Mean PR interval (ms) by day post-TAVI. (*A*) Displaced are the mean daily PR intervals as well as the percentage of patients with maximal PR interval per day. (*B*) Baseline, maximal, and pre-discharge PR intervals, *P* < 000.1 for all comparisons.

Of the 146 patients with isolated PR prolongation, 136 (93%) demonstrated similar heart rates (difference of <20 b.p.m.) at baseline and maximal PR ECGs, while in 10, the heart rate was different by more than 20 b.p.m. (in 8, it was faster, while in 2, it was slower compared with the baseline ECG). *Figure 3A* provides mean daily PR changes as well as day of maximal PR interval. The day post-TAVI with maximal PR prolongation was 1.1 ± 1.7 (median 0, IQR 0–2). Most patients (55.5%) reached maximal PR on the day of the TAVI procedure and 85% within 2 days post-TAVI. PR interval returned to 20 ms or less from baseline in 82 (56.2%) patients and was still prolonged on pre-discharge ECG compared with baseline in 64 (43.8%) patients [the difference between baseline and pre-discharge PR intervals as well as maximal and pre-discharge PR intervals was significant, *P* < 000.1 for all comparisons (*Figure 3B*)]. PR interval returned to 20 ms or less from baseline in 74 (50.7%) patients within 3 days post the procedure.

In addition, 115 (79%) out of the 146 patients with PR prolongation had 2 or more ECG during the 1 year period prior to the TAVI procedure. A change in PR interval of more than 20 ms, prior to the TAVI procedure, could be demonstrate in only 2 (1.7%) patients, while in 113, there was no change in PR.

### Comparison between patients with and without PR prolongation

Patients with isolated PR prolongation had higher rates of DM and lower rates of IHD (*Table 1*). In addition, rate of regular access through the femoral artery compared with alternative accesses was higher. On echocardiographic examination, their EF was more often ≥50% and their calculated aortic valve area was slightly larger. Their baseline PR interval was shorter with a mean delta PR prolongation of 40.2 ± 22 ms (median 32, IQR 26–44 ms). Other parameters, as well as rates of PPI or mortality, were not different between the groups.

### Separate comparison between patients with and without PR prolongation and narrow or wide QRS

Among patients with narrow QRS, those with post-procedural PR prolongation showed lower rates of IHD and EF less often below 50% compared with those without. Their baseline PR interval was shorter and their maximal PR was longer. In addition, no difference in rates of 1 year PPI and overall mortality or the combined rates of 1 year mortality and PPI was noted (*Table 2*). Similar comparison among patients with QRS width ≥ 120 demonstrated that patients with PR prolongation had higher rates of hyperlipidaemia as well as larger calculated aortic valve area and a tendency for shorter baseline PR interval. Other parameters and clinical outcomes (rates of PPI or mortality) were comparable (see Supplementary material online, *Table S2*). Mean delta PR prolongation was 39.2 ± 22 ms (median 32, IQR 26–44 ms) and 43.7 ± 24 ms (median 36, IQR 24–58 ms) among patients with narrow and wide QRS, respectively.

**Table 2:** Comparison of patients with narrow QRS with and without PR prolongation

	Isolated PR prolongation	No PR prolongation	*P* value
	*n* = 113	*n* = 215	
Age	79 ± 8.9	80.3 ± 8.1	0.32
Sex, female	59 (52.2)	105 (48.8)	0.561
Hyperlipidaemia	63 (55.8)	111 (54.4)	0.818
DM	49 (43.4)	77 (35.8)	0.182
HTN	97 (85.8)	167 (77.7)	0.076
IHD	44 (38.9)	118 (54.9)	**0.006**
Echocardiography			
LVEF^a^			
LVEF ≥ 50%	102 (90.3)	172 (80)	**0**.**017**
AVA	0.63 ± 0.14	0.59 ± 0.14	**0**.**018**
Peak gradient	81.3 ± 25.3	81.5 ± 23.5	0.937
Mean gradient	48.4 ± 17	47.3 ± 14.9	0.911
Medications			
Beta-blockers	48 (42.5)	102 (47.4)	0.391
Ca blockers	2 (1.8)	7 (3.3)	0.347
Amiodarone	3 (2.7)	14 (6.5)	0.105
ECG			
PR interval baseline	177.3 ± 32.7	184.5 ± 36.2	**<0**.**001**
PR interval > 200 ms^b^	70 (61.9)	54 (25.1)	**<0**.**001**
PR interval ≥ 240 ms^b^	23 (20.4)	21 (9.8)	**0**.**008**
Maximal PR	216.6 ± 40	184.5 ± 36.2	**<0**.**001**
Heart rate baseline	69.1 ± 10.8	71.4 ± 12.7	0.607
Baseline QRS interval	91.5 ± 11.8	90.6 ± 12.5	0.602
Valve type			
Sapien/Sapien XT/Sapien 3	51 (45.1)	110 (51.2)	0.110
CoreValve/Evolut R/Evolut Pro/Evolut Pro Plus	37 (32.7)	45 (20.9)	
Portico/Navitor	0 (0)	1 (0.5)	
Acurate Neo I/Acurate Neo II	25 (22.1)	59 (27.4)	
Hospitalization duration	4 (3–6)	4 (3–6)	0.344
Femoral approach	112 (99.1)	200 (93)	**0**.**015**
In-hospital PPI	0 (0)	2 (0.9)	0.304
1 year PPI	0 (0)	4 (1.8)	0.183
Overall PPI	2 (1.8)	7 (3.3)	0.434
1 year mortality	4 (3.5)	12 (5.6)	0.415
Overall mortality	28 (24.8)	58 (27)	0.667
1 year mortality or PPI	4 (3.5)	16 (7.4)	0.160
FU time, days	1045 (713.25–1849)	1067 (616–1900)	0.485

AVA, aortic valve area; DM, diabetes mellitus; FU, follow-up; HTN, hypertension; IHD, ischaemic heart disease; IVCD, intraventricular conduction disturbance; LBBB, left bundle branch block; LVEF, left ventricular ejection fraction; PPI, permanent pacemaker implantation; RBBB, right bundle branch block.

The bold means that it is statistically significant.

^a^Full distribution is presented in Supplementary material online, *Table S1*.

^b^Any PR either at baseline or maximal PR.

### Comparison of patients with PR ≥ 240 ms vs. shorter duration

As PR interval ≥ 240 is considered higher risk,^7,10^ we compared patients who showed these values (either at baseline or on maximal PR interval ECG) with patients who exhibited shorter PR interval duration. Patients with narrow QRS and PR interval ≥ 240 ms were more often males, and their QRS was wider. Their 1 year mortality rate was higher with no difference in pacemaker rates (see Supplementary material online, *Table S3*). Adding PR interval ≥ 240 to all multivariate analyses described below showed that it did not change the models nor remained statistically significant. No difference in mortality was observed among patients with wide QRS and PR interval ≥ 240 compared with those with PR interval < 240 ms nor with rates of PPI at 1 year follow-up (see Supplementary material online, *Table S4*).

### Cardiac implantable electronic device implantation and mortality during the first year post-transcatheter aortic valve implantation

Overall, 11 patients underwent cardiac implantable electronic device (CIED) implantation during the first year post-TAVI, 4 (2.7%) from the study group and 7 (2.4%) from the control group (*Table 3*). Most (6/11) occurred before discharge from the index hospitalization for the TAVI procedure. Of note, among the study group, no patient with baseline narrow QRS underwent CIED implantation during 1 year follow-up post-TAVI. Data on EPS results, which was conducted in four and three patients from the study and control groups, respectively, all with baseline wide QRS are provided in Supplementary material online, *Table S5*. Supplementary material online, *Table S6* describes 1 year mortality events and its cause. As AVB may present clinically as sudden cardiac death and as the cause of death was unknown in a sizeable number of patients, we further analysed in the next sections the combined outcome of CIED implantation and total mortality. Of note, only one patient from the study group with narrow QRS expired 60 days post-TAVI from unknown cause; all others had non-cardiac mode of death.

**Table 3:** Implanted CIED during the first year post-TAVI

	Baseline QRS	Timing of CIED^a^	Before/after discharge	Indication	CIED type
Study group					
#1	RBBB right axis	4	Before discharge	AVB	PM
#2	RBBB right axis	5	After discharge	Syncope	PM
#3	RBBB + LAHB	344	After discharge	LV dysfunction	CRTD
#4	LBBB	6	Before discharge	HV 80 ms on EPS and LV dysfunction	CRTD
Control group					
#1	Narrow QRS^b^	347	After discharge	Syncope and change in QRS to RBBB + LAHB	PM
#2	Narrow QRS	127	After discharge	Sick sinus syndrome	PM
#3	Narrow QRS	10	Before discharge	AVB	PM
#4	LBBB	139	After discharge	AVB	MICRA AV
#5	RBBB + LAHB	1	Before discharge	AVB	PM
#6	Narrow QRS	2	Before discharge	AVB	PM
#7	RBBB + LAHB	6	Before discharge	HV 85 ms on EPS	PM

AVB, atrio-ventricular block; CIED, cardiac implantable electronic device; CRTD, cardiac resynchronization therapy with defibrillator; EPS, electrophysiological study; LAHB, left anterior haemiblock; LBBB, left bundle branch block; LV, left ventricle; PM, dual-chamber pacemaker implantation; RBBB, right bundle branch block; TAVI, transcatheter aortic valve implantation.

^a^Days post-TAVI.

^b^This patient had narrow QRS on all ECGs during hospitalization and wide QRS on ECG performed ∼1 year post-discharge.

### Multivariate analysis for the combined endpoint of permanent pacemaker implantation and mortality

A multivariate backward stepwise Cox regression analysis demonstrated that age, baseline QRS width, and EF < 50% were significantly associated with the occurrence of the combined endpoint of death and/or PPI during follow-up (*Table 4*). Having isolated PR prolongation (study group) did not affect the combined clinical outcome. Separate multivariate analysis for patients with narrow QRS showed similar results. Both age and EF < 50% affected the combined outcome, while isolated PR prolongation did not. Analysing only patients with wide QRS demonstrated that male sex, baseline PR interval, as well as baseline QRS width were significantly associated with the combined endpoint, while DM was protective.

**Table 4:** Multivariate backward stepwise Cox regression analysis for the combined endpoint of PPI and mortality

	HR	95 CI	*P* value
All			
Study group	0.920	0.640–1.324	0.653
Age	1.042	1.017–1.068	**<0**.**001**
Baseline QRS width, ms	1.007	1.001–1.014	**0**.**034**
EF ≥ 50%	0.595	0.403–0.879	**0**.**009**
Narrow QRS			
Study group	0.857	0.545–1.348	0.505
Age	1.047	1.014–1.080	**0**.**005**
EF ≥ 50	0.454	0.278–0.743	**0**.**002**
Wide QRS			
Study group	1.562	0.786–3.106	0.203
Age	1.043	0.998–1.900	0.062
Sex, female	0.488	0.250–0.952	**0**.**035**
DM	0.387	0.185–0.808	**0**.**011**
Baseline PR, ms	1.008	1.001–1.016	**0**.**044**
Baseline QRS width, ms	1.016	1.000–1.033	**0**.**032**

CI, confidence interval; DM, diabetes mellitus; EP, ejection fraction; HR, hazard ratio; PPI, permanent pacemaker implantation.

The bold means that it is statistically significant.

A separate multivariate Cox regression analysis using maximal PR interval instead of baseline PR interval showed identical results for the whole group as well as for patients with narrow QRS (see Supplementary material online, *Table S7*). PR prolongation was not associated with clinical outcome. Among patients with wide QRS, QRS width was associated with the combined outcome, while DM was protective. The presence of RBBB showed a trend towards association with the combined outcome.

**Figure 4:**
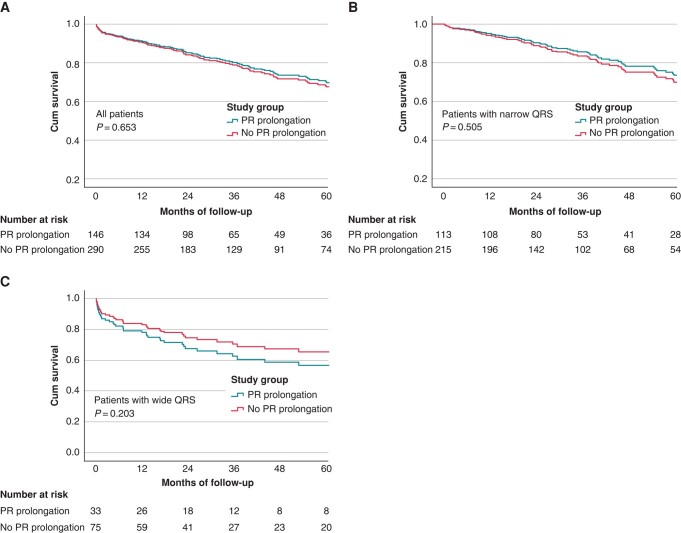
Adjusted survival curves demonstrating no difference in the occurrence of the combined outcome of mortality or pacemaker implantation in patients with and without PR prolongation. (*A*) All patients, (*B*) patients with narrow QRS, and (*C*) patients with wide QRS.

Adjusted survival curves demonstrating no difference in the occurrence of the combined outcome in patients with and without PR prolongation in all patients and separately in patients with narrow and wide QRS are presented in *Figure 4*.

### Multivariate analysis for mortality

A multivariate backward stepwise Cox regression analysis demonstrated that age and EF < 50% were significantly associated with mortality among the whole group and patients with narrow QRS, while age and male sex were associated with mortality among patients with wide QRS (see Supplementary material online, *Table S8*). Again, having isolated PR prolongation (study group) did not affect the mortality outcome. Also, using maximal PR instead of baseline PR in the multivariate Cox regression analysis did not change the results in any of the analyses.

Adjusted survival curves demonstrating no difference in the occurrence of the mortality in patients with and without PR prolongation in all patients and separately in patients with narrow and wide QRS are presented in Supplementary material online, *Figure S1*.

Due to low events, a multivariate analysis for the occurrence of PPI was not conducted; however, survival curves demonstrating no difference in the occurrence of PPI between patients with and without isolated PR prolongation are presented in Supplementary material online, *Figure S2*.

## Discussion

### Main findings

Our study included 436 patients who underwent TAVI, had a clear ECG before and after the procedure, and had no QRS prolongation after the procedure. Isolated PR prolongation of 40.2 ± 22 ms may occur in ∼13% of patients undergoing TAVI, with maximal prolongation occurring most commonly at the day of the procedure and in >90% of cases within 3 days. Among patients with narrow QRS, post-TAVI isolated PR prolongation and baseline or maximal PR interval values were not found to be associated with an increased risk for PPI or mortality. However, among patients with baseline wide QRS, a signal for an association between baseline PR interval and the combined clinical outcome (PPI and/or mortality) was found and should be further studied.

### Isolated PR prolongation

Most cases of PR prolongation were seen at similar heart rates as the baseline ECG suggesting it was related to the procedure rather than autonomic effect on the AV node. Moreover, most patients did not exhibit PR prolongation in multiple ECGs prior to TAVI, further supporting this notion. PR prolongation without a change in QRS may occur at either the AV node or the His bundle levels. The benign clinical effect of this phenomenon especially in patients with narrow QRS suggests that the effect occurs mainly at the AV node level. Regardless of the mechanism, our main purpose was to study the extent of the phenomenon and its clinical implication as will be further discussed in the next sections.

### Previous studies

Several studies demonstrated that TAVI may be associated with PR and QRS prolongation, and on EPS post-TAVI prolongation of either, AH, HV, and even intra-His intervals were shown.^2,15–18^ In addition, PR prolongation and/or prolonged PR interval were suggested as a risk factor for PPI.^19^ De Carlo *et al.*^20^ studied 275 consecutive post-TAVI patients. Permanent pacemaker implantation was associated with baseline RBBB, LAHB, and prolonged PR interval. Likewise, Mangieri *et al.*^21^ demonstrated that delta PR and baseline RBBB were independent predictors of advance heart block late (≥48 h) after TAVI. A meta-analysis including 11 210 TAVI patients from 41 studies suggested increased post-TAVI pacemaker risk in patients with baseline first-degree AVB, LAHB, RBBB, and intraprocedural AVB.^22^ Finally, Castro-Mejia *et al.*^10^ and Jørgensen *et al.*^7^ who studied 344 and 467 TAVI patients, respectively, found that PR ≥ 240 ms and QRS ≥ 150 ms were associated with increased post-TAVI pacemaker risk. It should be emphasized that in all up-to-date studies, the effect of PR prolongation was studied in patients who had or might have had simultaneous QRS widening as well. Therefore, the additive risk of isolated PR prolongation on future PPI was unknown.

### Narrow QRS

Only a minority of patients from the above quoted studies had prolonged PR or PR prolongation and narrow QRS. Jørgensen *et al.* reported on 30 patients (6.3% of total cohort) with first-degree AVB and narrow QRS of whom 3 developed CAVB (only 1 of them had PR > 240 ms, while in 2, it was > 200 ms). Moreover, it is unknown whether the PR interval was dynamically changing and whether these patients had, in addition, QRS widening that did not go above 120 ms.^7^ Castro-Mejia *et al.*^10^ reported on 12 patients with new first-degree AVB ≥ 240 ms and narrow QRS. The clinical outcome of these patients was not specifically reported, and again, whether changes in QRS width also occurred is unknown. It should be noted that in both studies, only a limited number of ECGs were analysed: at baseline, immediately post-TAVI, and in one of them also at pre-discharge. Moreover, patients’ numbers were too small to draw any meaningful conclusion. The European guidelines on cardiac pacing recommended that patients with new LBBB and either PR ≥ 240 or QRS ≥ 150 QRS should undergo EPS or prolonged monitoring (IIa recommendation).^13^ The same management was recommended to patients with wide QRS and PR prolongation ≥ 20 ms but with IIb strength of the recommendation due to limited data. In the absence of reported literature data, recommendations for patients with narrow QRS and PR prolongation were not given. Thus, the current study adds to the current literature by showing that in patients with narrow QRS, isolated PR prolongation is not associated with higher PPI risk. Of note, in the current study, patients with narrow QRS and PR ≥ 240 did have increased mortality risk, but this was not related to PR prolongation nor remained significant in a multivariate analysis. Nevertheless, despite being the largest literature report on this patient subgroup, the absolute number of patients with narrow QRS and PR ≥ 240 is relatively small (*n* = 44) and further validation is needed.

### Wide QRS

The current study involves a limited number of patients with baseline wide QRS and isolated PR prolongation (i.e. without QRS prolongation). QRS morphology distributed almost evenly between RBBB and LBBB with a minority having IVCD. Multivariate analysis suggested that baseline prolonged PR interval and baseline QRS width, rather than PR prolongation, are associated with the combined endpoint of mortality and PPI. It should be emphasized that the current study is not powered to give any strong recommendation regarding patients with baseline wide QRS but rather implies a potential higher risk among patients with wide QRS and prolonged baseline PR interval. In the current study, 10% (*n* = 5/50) of patients with baseline RBBB underwent PPI, two of them did not have PR prolongation, compared with 4.2% (*n* = 2/48) of patients with baseline LBBB. In addition, in the multivariate model using maximal PR, RBBB morphology tended to affect the combined outcome (*P* = 0.07). It seems that undergoing TAVI with baseline LBBB without changes in QRS width is associated with a good prognosis, while undergoing TAVI with baseline RBBB is associated with higher risk for AVB within 1 year, with and without QRS/PR prolongation. Current guidelines recommend that patients with baseline LBBB or IVCD and PR prolongation be managed with EPS or prolonged monitoring, while a PM implantation is recommended in patients with baseline RBBB and PR prolongation.^11–13^ However, a newer study among patients with RBBB did not demonstrate an association between PR changes and risk of delayed AVB, suggesting that better risk stratification tools are needed in this patient population.^23^

### Clinical implications

Currently, both QRS widening and PR prolongation are perceived as significant risk factors for the development of post-TAVI CAVB with no clear distinction regarding the risk in cases with isolated PR prolongation.^19^ The current study is the first to show that PR prolongation in patients with narrow QRS is associated with benign prognosis and may offer early patient mobilization from intensive to less strict in-hospital supervision or even earlier patient discharge.

### Limitations

An inherent limitation is the study design: a single centre observational retrospective data collection that spans over many years including older and newer valve designs. Nevertheless, the inclusion of all available patients prevented selection bias and our aim was to study the effect of the end result (i.e. isolated PR prolongation) on clinical outcome. In addition, despite being, to our knowledge, the largest reported cohort on patients with isolated PR prolongation and narrow QRS, the number of patients with narrow QRS and PR ≥ 240 is not extensive and should be further validated. The mode of death was not known in many patients, and therefore, we used the combined endpoint of mortality and PPI to demonstrate comparable outcome between patients with and without PR prolongation. It should be noted that only one patient from the study group with narrow QRS expired from unknown cause; all others had non-cardiac death, further supporting the benign effect of isolated PR prolongation among this patient group. In addition, the main study conclusion is related to patients with baseline narrow QRS and the effect of isolated PR prolongation in different subgroups of patients with wide QRS deserves further study.

## Conclusions

Post-TAVI patients with isolated PR prolongation and baseline narrow QRS demonstrate a benign clinical course that is similar to patients who do not exhibit post-TAVI ECG conduction disturbances.

## Supplementary Material

euae011_Supplementary_Data

## Data Availability

The data from this study are available from the corresponding author, upon appropriate and reasonable request.

## References

[1] Urena M , Mok M, Serra V, Dumont E, Nombela-Franco L, DeLarochellière R et al Predictive factors and long-term clinical consequences of persistent left bundle branch block following transcatheter aortic valve implantation with a balloon-expandable valve. J Am Coll Cardiol 2012;60:1743–52. 23040577 10.1016/j.jacc.2012.07.035

[2] Tovia-Brodie O , Ben-Haim Y, Joffe E, Finkelstein A, Glick A, Rosso R et al The value of electrophysiologic study in decision-making regarding the need for pacemaker implantation after TAVI. J Interv Card Electrophysiol 2017;48:121–30. 27987072 10.1007/s10840-016-0218-2

[3] Rodes-Cabau J , Urena M, Nombela-Franco L, Amat-Santos I, Kleiman N, Munoz-Garcia A et al Arrhythmic burden as determined by ambulatory continuous cardiac monitoring in patients with new-onset persistent left bundle branch block following transcatheter aortic valve replacement: the MARE study. JACC Cardiovasc Interv 2018;11:1495–505. 30031719 10.1016/j.jcin.2018.04.016

[4] Zito A , Princi G, Lombardi M, D'Amario D, Vergallo R, Aurigemma C et al Long-term clinical impact of permanent pacemaker implantation in patients undergoing transcatheter aortic valve implantation: a systematic review and meta-analysis. Europace 2022;24:1127–36. 35138367 10.1093/europace/euac008 PMC9460982

[5] Yagel O , Belhassen B, Planer D, Amir O, Elbaz-Greener G. The R-wave amplitude in V1 on baseline electrocardiogram correlates with the occurrence of high-degree atrioventricular block following left bundle branch block after transcatheter aortic valve replacement. Europace 2023;25:euad066. 36938963 10.1093/europace/euad066 PMC10227649

[6] Schoechlin S , Jalil F, Blum T, Ruile P, Hein M, Nührenberg TG et al Need for pacemaker implantation in patients with normal QRS duration immediately after transcatheter aortic valve implantation. Europace 2019;21:1851–6. 31578544 10.1093/europace/euz261

[7] Jorgensen TH , De Backer O, Gerds TA, Bieliauskas G, Svendsen JH, Sondergaard L. Immediate post-procedural 12-lead electrocardiography as predictor of late conduction defects after transcatheter aortic valve replacement. JACC Cardiovasc Interv 2018;11:1509–18. 30093055 10.1016/j.jcin.2018.04.011

[8] Massoullie G , Ploux S, Souteyrand G, Mondoly P, Pereira B, Amabile N et al Incidence and management of atrioventricular conduction disorders in new-onset left bundle branch block after TAVI: a prospective multicenter study. Heart Rhythm 2023;20:699–706. 36646235 10.1016/j.hrthm.2023.01.013

[9] Nazif TM , Chen S, George I, Dizon JM, Hahn RT, Crowley A et al New-onset left bundle branch block after transcatheter aortic valve replacement is associated with adverse long-term clinical outcomes in intermediate-risk patients: an analysis from the PARTNER II trial. Eur Heart J 2019;40:2218–27. 31505615 10.1093/eurheartj/ehz227

[10] Castro-Mejia AF , Amat-Santos I, Ortega-Armas ME, Baz JA, Moreno R, Diaz JF et al Development of atrioventricular and intraventricular conduction disturbances in patients undergoing transcatheter aortic valve replacement with new generation self-expanding valves: a real world multicenter analysis. Int J Cardiol 2022;362:128–36. 35550389 10.1016/j.ijcard.2022.05.014

[11] Auffret V , Webb JG, Eltchaninoff H, Muñoz-García AJ, Himbert D, Tamburino C et al Clinical impact of baseline right bundle branch block in patients undergoing transcatheter aortic valve replacement. JACC Cardiovasc Interv 2017;10:1564–74. 28734885 10.1016/j.jcin.2017.05.030

[12] Watanabe Y , Kozuma K, Hioki H, Kawashima H, Nara Y, Kataoka A et al Pre-existing right bundle branch block increases risk for death after transcatheter aortic valve replacement with a balloon-expandable valve. JACC Cardiovasc Interv 2016;9:2210–6. 27832846 10.1016/j.jcin.2016.08.035

[13] Glikson M , Nielsen JC, Kronborg MB, Michowitz Y, Auricchio A, Barbash IM et al 2021 ESC guidelines on cardiac pacing and cardiac resynchronization therapy. Europace 2022;24:71–164. 34455427 10.1093/europace/euab232 PMC13179788

[14] Surawicz B , Childers R, Deal BJ, Gettes LS, Bailey JJ, Gorgels A et al AHA/ACCF/HRS recommendations for the standardization and interpretation of the electrocardiogram: part III: intraventricular conduction disturbances: a scientific statement from the American Heart Association Electrocardiography and Arrhythmias Committee, Council on Clinical Cardiology; the American College of Cardiology Foundation; and the Heart Rhythm Society: endorsed by the International Society for Computerized Electrocardiology. Circulation 2009;119:e235–40. 19228822 10.1161/CIRCULATIONAHA.108.191095

[15] Bourenane H , Galand V, Boulmier D, Leclercq C, Leurent G, Bedossa M et al Electrophysiological study-guided permanent pacemaker implantation in patients with conduction disturbances following transcatheter aortic valve implantation. Am J Cardiol 2021;149:78–85. 33753040 10.1016/j.amjcard.2021.03.014

[16] Rivard L , Schram G, Asgar A, Khairy P, Andrade JG, Bonan R et al Electrocardiographic and electrophysiological predictors of atrioventricular block after transcatheter aortic valve replacement. Heart Rhythm 2015;12:321–9. 25446155 10.1016/j.hrthm.2014.10.023

[17] Kostopoulou A , Karyofillis P, Livanis E, Thomopoulou S, Stefopoulos C, Doudoumis K et al Permanent pacing after transcatheter aortic valve implantation of a CoreValve prosthesis as determined by electrocardiographic and electrophysiological predictors: a single-centre experience. Europace 2016;18:131–7. 26060209 10.1093/europace/euv137

[18] Badertscher P , Knecht S, Spies F, Auberson C, Salis M, Jeger RV et al Value of periprocedural electrophysiology testing during transcatheter aortic valve replacement for risk stratification of patients with new-onset left bundle-branch block. J Am Heart Assoc 2022;11:e026239. 35876404 10.1161/JAHA.122.026239 PMC9375470

[19] Badertscher P , Knecht S, Zeljkovic I, Sticherling C, de Asmundis C, Conte G et al Management of conduction disorders after transcatheter aortic valve implantation: results of the EHRA survey. Europace 2022;24:1179–85. 35348646 10.1093/europace/euac027

[20] De Carlo M , Giannini C, Bedogni F, Klugmann S, Brambilla N, De Marco F et al Safety of a conservative strategy of permanent pacemaker implantation after transcatheter aortic CoreValve implantation. Am Heart J 2012;163:492–9. 22424022 10.1016/j.ahj.2011.12.009

[21] Mangieri A , Lanzillo G, Bertoldi L, Jabbour RJ, Regazzoli D, Ancona MB et al Predictors of advanced conduction disturbances requiring a late (>/=48 H) permanent pacemaker following transcatheter aortic valve replacement. JACC Cardiovasc Interv 2018;11:1519–26. 30093056 10.1016/j.jcin.2018.06.014

[22] Siontis GC , Juni P, Pilgrim T, Stortecky S, Büllesfeld L, Meier B et al Predictors of permanent pacemaker implantation in patients with severe aortic stenosis undergoing TAVR: a meta-analysis. J Am Coll Cardiol 2014;64:129–40. 25011716 10.1016/j.jacc.2014.04.033

[23] Isogai T , Dykun I, Agrawal A, Shekhar S, Tarakji KG, Wazni OM et al Evaluation of the 2021 European Society of Cardiology guidelines in pre-existing right bundle branch block patients undergoing transcatheter aortic valve implantation with a balloon-expandable valve. Eur Heart J Open 2022;2:oeac014. 35919121 10.1093/ehjopen/oeac014 PMC9242057

