# Supplementary material for: Post-transcatheter aortic valve implantation isolated PR prolongation: incidence and clinical significance
Source: Europace. 2024 Jan 16;26(1):euae011. doi: 10.1093/europace/euae011 (PMC10808043; doi:10.1093/europace/euae011)
Supplement: euae011_Supplementary_Data [file euae011_supplementary_data.zip › Sup Figures pr prolongation revision.pptx]

## Slide 1
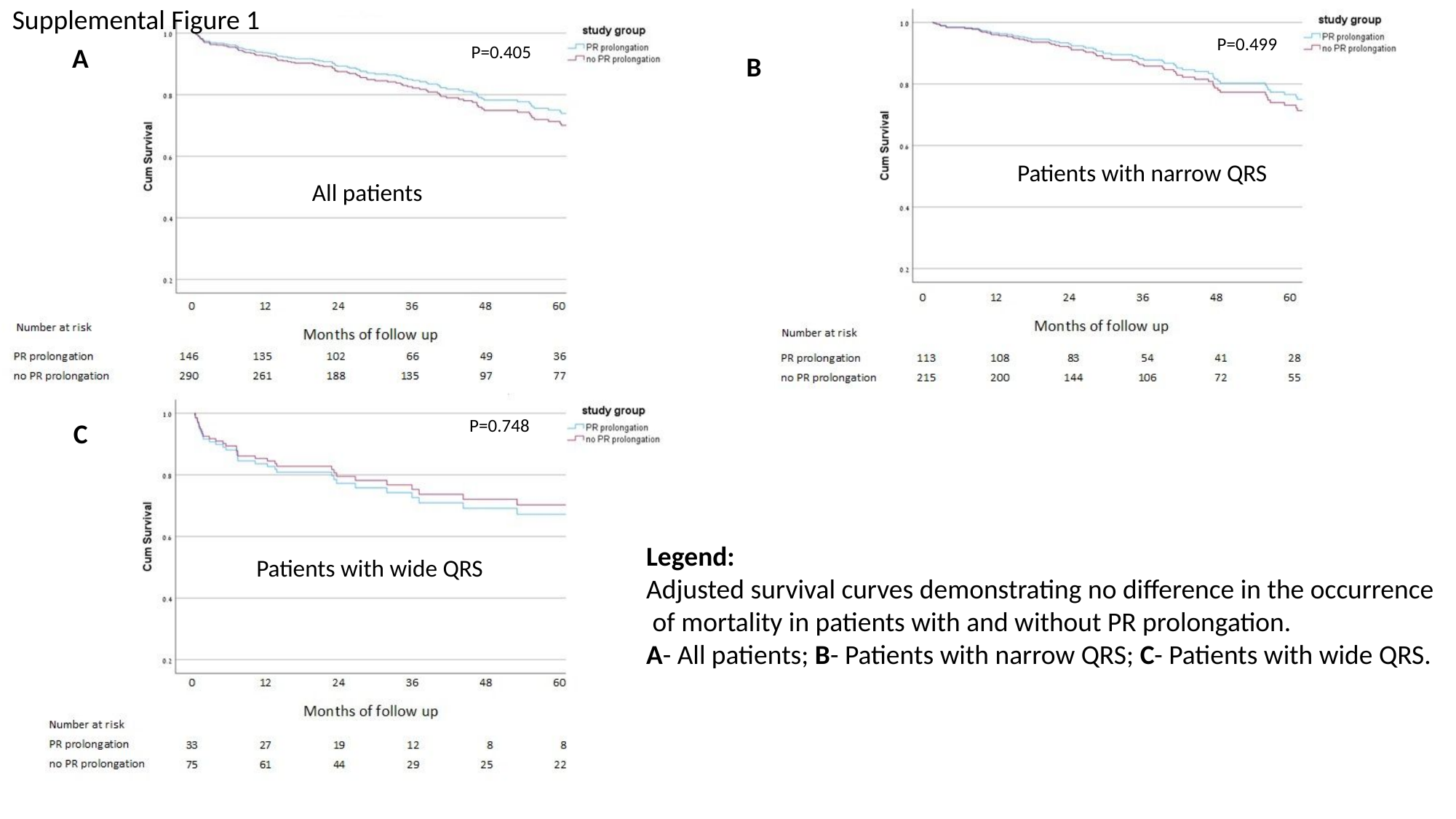

Supplemental Figure 1
P=0.499
A
P=0.405
A
B
Patients with narrow QRS
All patients
P=0.748
C
Legend:
Adjusted survival curves demonstrating no difference in the occurrence
 of mortality in patients with and without PR prolongation.
A- All patients; B- Patients with narrow QRS; C- Patients with wide QRS.
Patients with wide QRS

## Slide 2
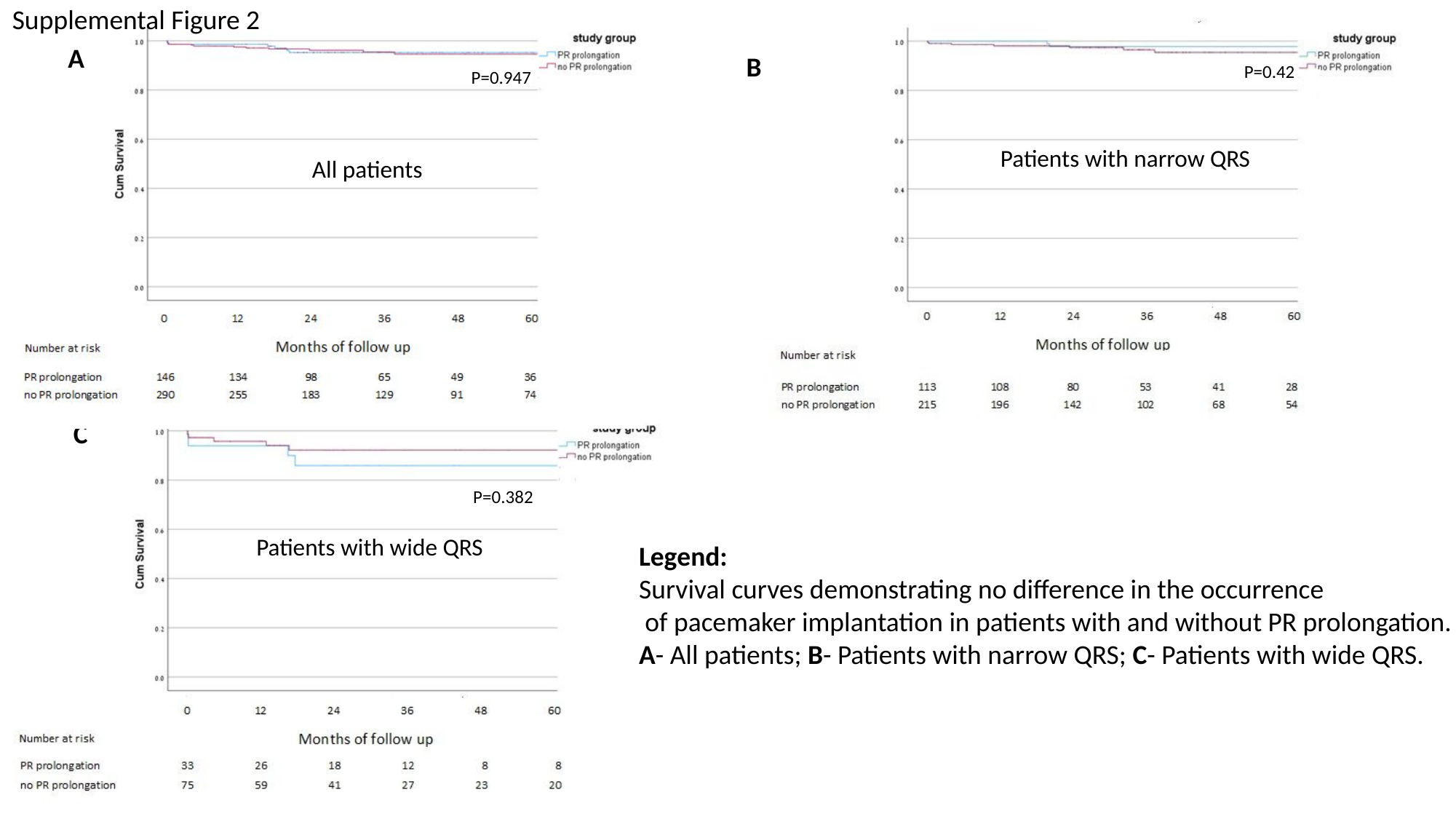

Supplemental Figure 2
A
B
P=0.42
P=0.947
Patients with narrow QRS
All patients
C
P=0.382
Patients with wide QRS
Legend:
Survival curves demonstrating no difference in the occurrence
 of pacemaker implantation in patients with and without PR prolongation.
A- All patients; B- Patients with narrow QRS; C- Patients with wide QRS.
