# Supplementary material for: Post-transcatheter aortic valve implantation isolated PR prolongation: incidence and clinical significance
Source: Europace. 2024 Jan 16;26(1):euae011. doi: 10.1093/europace/euae011 (PMC10808043; doi:10.1093/europace/euae011)
Supplement: euae011_Supplementary_Data [file euae011_supplementary_data.zip › Supplemental Tables PR prolongation revision.docx]

Supplemental Table 1: Distribution of ejection fraction

| All patients | Isolated PR prolongation | No PR prolongation | P value |
| --- | --- | --- | --- |
|  | n=146 | n=290 |  |
| **LVEF** |  |  |  |
| LVEF ≥ 50% | 123 (84.2) | 220 (75.9) | **0.044** |
| <30 | 1 (0.7) | 4 (1.4) | 0.358 |
| 30-35 | 4 (2.7) | 6 (2.1) |  |
| 35-39 | 4 (2.7) | 19 (6.6) |  |
| 40-44 | 7 (4.8) | 18 (6.2) |  |
| 45-49 | 7 (4.8) | 23 (7.9) |  |
| 50-54 | 24 (16.4) | 35 (12.1) |  |
| 55-70 | 99 (67.8) | 185 (63.8) |  |
|  |  |  |  |
| Patients with narrow QRS | Isolated PR prolongation | No PR prolongation | P value |
|  | n=113 | n=215 |  |
| **LVEF** |  |  |  |
| LVEF ≥ 50% | 102 (90.3) | 172 (80) | **0.017** |
| <30 | 0 (0) | 1 (0.5) | 0.221 |
| 30-35 | 1 (0.9) | 3 (1.4) |  |
| 35-39 | 2 (1.8) | 12 (5.6) |  |
| 40-44 | 5 (4.4) | 11 (5.1) |  |
| 45-49 | 3 (2.7) | 16 (7.4) |  |
| 50-54 | 21 (18.6) | 27 (12.6) |  |
| 55-70 | 81 (71.7) | 145 (67.4) |  |

LVEF=left ventricular ejection fraction

Supplemental Table 2: Comparison of patients with wide QRS with and without PR prolongation

| Patients with wide QRS | Isolated PR prolongation | No PR prolongation | P value |
| --- | --- | --- | --- |
|  | n=33 | n=75 |  |
| Age | 79.8 ± 9.5 | 82.1 ± 7.8 | 0.178 |
| Sex, female | 10 (30.3) | 36 (48) | 0.87 |
| Hyperlipidemia | 22 (66.7) | 32 (42.7) | **0.022** |
| DM | 17 (51.5) | 20 (26.7) | 0.012 |
| HTN | 26 (78.8) | 62 (82.7) | 0.633 |
| IHD | 14 (42.4) | 43 (57.3) | 0.153 |
| **Echocardiography** |  |  |  |
| **LVEF** |  |  |  |
| LVEF ≥ 50% | 21 (63.6) | 48 (64) | 0.971 |
| <30 | 1 (3) | 3 (4) | 0.924 |
| 30-35 | 3 (9.1) | 3 (4) |  |
| 35-39 | 2 (6.1) | 7 (9.3) |  |
| 40-44 | 2 (6.1) | 7 (9.3) |  |
| 45-49 | 4 (12.1) | 7 (9.3) |  |
| 50-54 | 3 (9.1) | 8 (10.7) |  |
| 55-70 | 18 (54.5) | 40 (53.3) |  |
| AVA | 0.7 ± 0.21 | 0.61 ± 0.15 | **0.025** |
| Peak gradient | 77.6 ± 22.7 | 72.3 ± 27.4 | 0.291 |
| Mean gradient | 44.1 ± 14.8 | 41.9 ± 17.3 | 0.515 |
| **Medications** |  |  |  |
| Beta blockers | 9 (27.3) | 28 (37.3) | 0.31 |
| Ca Blockers | 0 (0) | 1 (1.3) | 0.505 |
| Amiodarone | 4 (12.1) | 10 (13.3) | 0.863 |
| **ECG** |  |  |  |
| PR interval baseline | 185.6 ± 29.8 | 198.1 ± 37.3 | 0.067 |
| PR interval > 200 ms* | 24 (72.7) | 29 (38.7) | **0.001** |
| PR interval ≥ 240 ms* | 14 (42.4) | 10 (13.3) | **<0.001** |
| Maximal PR | 229.2 ± 39.5 | 198.1 ± 37.3 | **<0.001** |
| Heart rate baseline | 69.9 ± 15.2 | 71.3 ± 12.8 | 0.393 |
| Baseline QRS interval | 140 ± 16.9 | 140.4 ± 15.8 | 0.815 |
| QRS interval ≥ 120 ms | 33 (100) | 75 (100) | na |
| QRS interval ≥ 150 ms | 15 (45.5) | 19 (25.3) | 0.038 |
| RBBB | 16 (48.5) | 34 (45.3) | 0.282 |
| LBBB | 12 (36.4) | 36 (48) |  |
| IVCD | 5 (15.2) | 5 (6.7) |  |
| **Valve type** |  |  |  |
| Sapien/Sapien XT/Sapien 3 | 20 (60.6) | 41 (54.7) | 0.68 |
| CoreValve/Evolut R/ Evolut Pro/ Evolut Pro Plus | 7 (21.2) | 22 (29.3) |  |
| Portico/ Navitor | 0 (0) | 0 (0) |  |
| Acurate Neo I/ Acurate Neo II | 6 (18.2) | 12 (16) |  |
| Hospitalization duration | 4.5 [3-6.75] | 5 [3-7] | 0.754 |
| Femoral approach | 32 (97) | 72 (96) | 0.806 |
| In hospital PPI | 2 (6.1) | 2 (2.7) | 0.39 |
| 1-year PPI | 4 (12.1) | 3 (4) | 0.126 |
| Overall PPI | 4 (12.1) | 5 (6.7) | 0.345 |
| 1-year mortality | 6 (18.2) | 13 (17.3) | 0.915 |
| Overall mortality | 11 (33.3) | 29 (38.7) | 0.597 |
| 1-year mortality or PPI | 9 (27.3) | 15 (20) | 0.402 |
| FU time, days | 838 [419-1678.5] | 812 [453-2011] | 0.86 |

*Any PR either at baseline or maximal PR.

AVA= aortic valve area; DM=diabetes mellitus; FU=follow-up; HTN=hypertension; IHD=ischemic heart disease; IVCD=intraventricular conduction disturbance, LBBB=left bundle branch block; LVEF=left ventricular ejection fraction; PPI=permanent pacemaker implantation; RBBB=right bundle branch block.

Supplemental Table 3: Comparison or patients with narrow QRS with and without PR ≥ 240 ms

| Patients with narrow QRS | Any PR ≥240 | PR < 240 | P value |
| --- | --- | --- | --- |
|  | n=44 | n=284 |  |
| Age | 81.5 ± 6.3 | 79.6 ± 8.6 | 0.077 |
| Sex, female | 14 (31.8) | 150 (52.8) | **0.01** |
| Hyperlipidemia | 25 (56.8) | 155 (54.6) | 0.781 |
| DM | 18 (40.9) | 108 (38) | 0.715 |
| HTN | 38 (86.4) | 226 (79.6) | 0.291 |
| IHD | 22(50) | 140 (49.3) | 0.931 |
| **Echocardiography** |  |  |  |
| **LVEF** |  |  |  |
| LVEF ≥ 50% | 39 (88.6) | 235 (82.7) | 0.327 |
| <30 | 0 (0) | 1 (0.4) | 0.460 |
| 30-35 | 0 (0) | 4 (1.4) |  |
| 35-39 | 3 (6.6) | 11 (3.9) |  |
| 40-44 | 1 (2.3) | 15 (5.3) |  |
| 45-49 | 1 (2.3) | 18 (6.3) |  |
| 50-54 | 10 (22.7) | 38 (13.4) |  |
| 55-70 | 29 (65.9) | 197 (69.4) |  |
| AVA | 0.63 ± 0.12 | 0.6 ± 0.15 | 0.47 |
| Peak gradient | 79.6 ± 26.8 | 81.7 ± 23.7 | 0.912 |
| Mean gradient | 46.6 ± 16.3 | 47.8 ± 15.5 | 0.830 |
| **Medications** |  |  |  |
| Beta blockers | 16 (36.4) | 134 (47.2) | 0.180 |
| Ca Blockers | 3 (6.8) | 6 (2.1) | 0.106 |
| Amiodarone | 2 (4.5) | 15 (5.3) | 0.838 |
| **ECG** |  |  |  |
| PR interval baseline | 238.6 ± 36.2 | 173.3 ± 25.6 | **<0.001** |
| PR interval > 200 ms* | 44 (100) | 80 (28.2) | **<0.001** |
| PR interval ≥ 240 ms* | 44 (100) | 0 (0) | **<0.001** |
| Maximal PR | 269.2 ± 30 | 184.2 ± 28 | **<0.001** |
| Heart rate baseline | 71.1 ± 13.6 | 70.6 ± 11.9 | 0.607 |
| Baseline QRS interval | 97.1 ± 13.7 | 89.9 ± 11.7 | **<0.001** |
| **Valve type** |  |  |  |
| Sapien/Sapien XT/Sapien 3 | 21 (47.7) | 140 (49.3) | 0.715 |
| CoreValve/Evolut R/ Evolut Pro/ Evolut Pro Plus | 9 (20.5) | 73 (25.7) |  |
| Portico/ Navitor | 0 (0) | 1 (0.4) |  |
| Acurate Neo I/ Acurate Neo II | 14 (31.8) | 70 (24.6) |  |
| Hospitalization duration | 4 [3-7] | 4 [3-6] | 0.237 |
| Femoral approach | 42 (95.5) | 270 (95.1) | 0.912 |
| In hospital PPI | 1 (2.3) | 1 (0.4) | 0.251 |
| 1-year PPI | 1 (2.3) | 3 (1) | 0.440 |
| Overall PPI | 2 (4.5) | 7 (2.5) | 0.345 |
| 1-year mortality | 5 (11.4) | 11 (3.9) | **0.048** |
| Overall mortality | 14 (31.8) | 72 (25.4) | 0.364 |
| 1-year mortality or PPI | 6 (13.6) | 14 (4.9) | **0.037** |
| FU time, days | 1075 [527.25-1940.5] | 1067 [679-1867] | 0.757 |

*Any PR either at baseline or maximal PR. AVA= aortic valve area; DM=diabetes mellitus; FU=follow-up; HTN=hypertension; IHD=ischemic heart disease; IVCD=intraventricular conduction disturbance, LBBB=left bundle branch block; LVEF=left ventricular ejection fraction; PPI= permanent pacemaker implantation; RBBB=right bundle branch block.

Supplemental Table 4: Comparison or patients with wide QRS with and without PR ≥ 240 ms

| Patients with wide QRS | Any PR ≥240 | PR < 240 | P value |
| --- | --- | --- | --- |
|  | n=24 | n=84 |  |
| Age | 81.5 ± 8.7 | 81.4 ± 8.4 | 0.94 |
| Sex, female | 10 (41.7) | 36 (42.9) | 0.917 |
| Hyperlipidemia | 12 (50) | 42 (50) | 1 |
| DM | 6 (25) | 31 (36.9) | 0.278 |
| HTN | 20 (83.3) | 68 (81) | 0.791 |
| IHD | 10 (41.7) | 47 (56) | 0.216 |
| **Echocardiography** |  |  |  |
| **LVEF** |  |  |  |
| LVEF ≥ 50% | 15 (62.5) | 54 (64.3) | 0.872 |
| <30 | 0 (0) | 4 (4.8) | 0.442 |
| 30-35 | 0 (0) | 6 (7.1) |  |
| 35-39 | 4 (16.7) | 5 (6) |  |
| 40-44 | 2 (8.3) | 7 (8.3) |  |
| 45-49 | 3 (12.5) | 8 (9.5) |  |
| 50-54 | 3 (12.5) | 8 (9.5) |  |
| 55-70 | 12 (50) | 46 (54.8) |  |
| AVA | 0.61 ± 0.13 | 0.65 ± 0.19 | 0.424 |
| Peak gradient | 76.1 ± 27.4 | 73.3 ± 25.8 | 0.399 |
| Mean gradient | 42.6 ± 16.8 | 42.6 ± 16.6 | 0.727 |
| **Medications** |  |  |  |
| Beta blockers | 4 (16.7) | 33 (39.3) | **0.039** |
| Ca Blockers | 0 (0) | 1 (1.2) | 0.778 |
| Amiodarone | 5 (20.8) | 9 (10.7) | 0.168 |
| **ECG** |  |  |  |
| PR interval baseline | 233.7 ± 38.9 | 183 ± 25 | **<0.001** |
| PR interval > 200 ms | 24 (100) | 29 (34.5) | **<0.001** |
| PR interval ≥ 240 ms | 24 (100) | 0 (0) | **<0.001** |
| Maximal PR | 268.3 ± 20 | 190.3 ± 25 | **<0.001** |
| Heart rate baseline | 70 ± 12.3 | 71.1 ± 14 | 0.607 |
| Baseline QRS interval | 97.1 ± 13.7 | 89.8 ± 11.7 | **<0.001** |
| QRS interval ≥ 120 ms* | 24 (100) | 84 (100) | na |
| QRS interval ≥ 150 ms* | 10 (41.7) | 24 (28.6) | 0.223 |
| RBBB | 14 (58.3) | 36 (42.9) | 0.396 |
| LBBB | 8 (33.3) | 40 (47.6) |  |
| IVCD | 2 (8.3) | 8 (9.5) |  |
| **Valve type** |  |  |  |
| Sapien/Sapien XT/Sapien 3 | 14 (58.3) | 47 (56) | 0.818 |
| CoreValve/Evolut R/ Evolut Pro/ Evolut Pro Plus | 7 (29.2) | 22 (26.2) |  |
| Portico/ Navitor | 0 (0) | 0 (0) |  |
| Acurate Neo I/ Acurate Neo II | 3 (12.5) | 15 (17.9) |  |
| Hospitalization duration | 6 [3-8] | 4 [3-7] | 0.068 |
| Femoral approach | 23 (95.8) | 81 (96.4) | 0.64 |
| In hospital PPI | 2 (8.3) | 2 (2.4) | 0.213 |
| 1-year PPI | 3 (12.5) | 4 (4.8) | 0.182 |
| Overall PPI | 4 (16.7) | 5 (6) | 0.108 |
| 1-year mortality | 4 (16.7) | 15 (17.9) | 0.581 |
| Overall mortality | 9 (37.5) | 31 (28.7) | 0.958 |
| 1-year mortality or PPI | 6 (25) | 18 (21.4) | 0.711 |
| FU time, days | 978 [449.25-2082.25] | 796 [432-1931.75] | 0.478 |

*Any PR either at baseline or maximal PR.

AVA= aortic valve area; DM=diabetes mellitus; FU=follow-up; HTN=hypertension; IHD=ischemic heart disease; IVCD=intraventricular conduction disturbance, LBBB=left bundle branch block; LVEF=left ventricular ejection fraction; PPI= permanent pacemaker implantation; RBBB=right bundle branch block.

Supplemental Table 5: EPS data

|  | Baseline QRS | Baseline PR | QRS width | Maximal PR | Day | PR during EPS | Day of EPS | AH | HV | PR before discharge | Day of PPI | ** |
| --- | --- | --- | --- | --- | --- | --- | --- | --- | --- | --- | --- | --- |
| **Study group** |  |  |  |  |  |  |  |  |  |  |  |  |
|  | LBBB | 210 | 145 | 300 | 3 | 300 | 3 | 160 | 50 | 300 | - |  |
|  | RBBB | 180 | 160 | 290 | 5 | 290 | 5 | 220 | 65 | 290 | - |  |
|  | RBBB + LAHB | 176 | 140 | 230 | 3 | 190 | 2 | 97 | 65 | 230 | 344 | #3 |
|  | LBBB | 214 | 170 | 270 | 2 | 270 | 2 | 174 | 80 | 270* | 6 | #4 |
| **Control group** |  |  |  |  |  |  |  |  |  |  |  |  |
|  | RBBB + LAHB | 179 | 154 |  |  |  | 5 | 95 | 85 |  | 6 | #7 |
|  | RBBB + LAHB | 172 | 160 |  |  |  | 5 | 88 | 61 |  | - |  |
|  | LBBB | 230 | 150 |  |  |  | 4 | 138 | 58 |  | - |  |

A prolonged AH interval was seen in all study group patients except 1 who normalized the PR interval before the EPS, while HV interval > 70 ms was seen in 1 patient from each group.

All measurements are in ms.

* Last PR before pacing

** Patient number in Table 3

AH= atrio-His interval; EPS= electrophysiological study; HV= His-ventricular interval; LAHB= left anterior hemi block; LBBB=left bundle branch block; PPI= pacemaker implantation; RBBB=right bundle branch block.

Supplemental Table 6: 1-year mortality data.

|  | Age | Sex | Baseline QRS | Day of event | Cause |
| --- | --- | --- | --- | --- | --- |
| **Study group** | 88 | Male | LBBB | 22 | Infection |
|  | 86 | Male | LBBB | 38 | Infection |
|  | 86 | Female | RBBB | 212 | Unknown |
|  | 88 | Female | RBBB | 108 | Unknown |
| * | 87 | Male | RBBB | 214 | Infection |
|  | 50 | Male | LBBB | 27 | Unknown |
|  | 78 | Male | Narrow QRS | 344 | AHF |
|  | 94 | Female | Narrow QRS | 41 | Infection |
|  | 80 | Female | Narrow QRS | 206 | Hip fracture |
|  | 66 | Male | Narrow QRS | 60 | Unknown |
|  |  |  |  |  |  |
| **Control group** | 82 | Male | LBBB | 0^ | AI and AHF |
|  | 88 | Female | RBBB | 33 | Infection |
|  | 88 | Female | LBBB | 219 | Hypoxia^^ |
|  | 95 | Female | LBBB | 2^&^ | Unknown |
|  | 71 | Male | RBBB | 157 | Unknown |
|  | 86 | Female | RBBB | 73 | Unknown |
|  | 88 | Male | LBBB | 244 | COPD |
|  | 78 | Male | LBBB | 218 | Unknown |
|  | 86 | Male | RBBB | 142 | Malignancy |
|  | 87 | Female | LBBB | 143 | AHF |
| ** | 74 | Male | LBBB | 309 | Infection |
|  | 86 | Male | RBBB | 20 | CVA |
|  | 92 | Male | RBBB | 12 | Unknown |
|  | 88 | Male | Narrow QRS | 167 | Infection |
|  | 80 | Male | Narrow QRS | 73 | Infection |
|  | 93 | Male | Narrow QRS | 300 | Unknown |
|  | 82 | Female | Narrow QRS | 302 | Malignancy |
|  | 93 | Male | Narrow QRS | 44 | Infection |
|  | 84 | Male | Narrow QRS | 252 | Infection |
|  | 89 | Male | Narrow QRS | 279 | Infection |
|  | 87 | Male | Narrow QRS | 238 | Malignancy |
|  | 100 | Female | Narrow QRS | 42 | Unknown |
|  | 82 | Male | Narrow QRS | 24 | Infection |
|  | 74 | Female | Narrow QRS | 71 | Unknown |
|  | 78 | Male | Narrow QRS | 10 | Unknown |

*Also received a PM on day 4 post-TAVI (study group patient #1 in Table 3). **Also received a PM on day 139 post-TAVI (control group patient #4 in Table 3). ^Died in the cardiac intensive care unit several hours after the procedure. ^^Found in severe hypoxia and died during asystolic resuscitation. ^&^Died after discharge.

AHF=acute heart failure; AI= aortic insufficiency; COPD= chronic obstructive pulmonary disease; CVA= cerebrovascular accident; LBBB=left bundle branch block; PM= pacemaker; RBBB=right bundle branch block.

Supplemental Table 7: multivariate backward stepwise cox regression analysis for the combined endpoint of PPI and mortality

|  | **HR** | **95 CI** | **p value** |
| --- | --- | --- | --- |
| **All** |  |  |  |
| Study group | 0.920 | 0.640-1.324 | 0.653 |
| Age | 1.042 | 1.017-1.068 | **<0.001** |
| Baseline QRS width, ms | 1.007 | 1.001-1.014 | **0.034** |
| EF ≥ 50% | 0.595 | 0.403-0.879 | **0.009** |
| **Narrow QRS** |  |  |  |
| Study group | 0.857 | 0.545-1.348 | 0.505 |
| Age | 1.047 | 1.014-1.080 | **0.005** |
| EF ≥ 50% | 0.454 | 0.278-0.743 | **0.002** |
| **Wide QRS** |  |  |  |
| Study group | 1.342 | 0.698-2.578 | 0.378 |
| Age | 1.043 | 0.994-1.094 | 0.085 |
| Sex, female | 0.533 | 0.266-1.068 | 0.076 |
| DM | 0.369 | 0.177-0.771 | **0.008** |
| Baseline QRS width, ms | 1.018 | 1.000-1.035 | **0.044** |
| Baseline RBBB | 1.777 | 0.950-3.325 | 0.072 |

CI=confidence interval; DM=diabetes mellitus; EP=ejection fraction; HR=hazard ratio; PPI=permanent pacemaker implantation; RBBB=right bundle branch block.

Supplemental Table 8: multivariate backward stepwise cox regression analysis for mortality

|  | **HR** | **95 CI** | **p value** |
| --- | --- | --- | --- |
| **All** |  |  |  |
| Study group | 0.850 | 0.579-1.247 | 0.405 |
| Age | 1.053 | 1.025-1.082 | **<0.001** |
| EF ≥ 50% | 0.533 | 0.362-0.783 | **0.001** |
| **Narrow QRS** |  |  |  |
| Study group | 0.852 | 0.535-1.357 | 0.499 |
| Age | 1.054 | 1.020-1.089 | **0.002** |
| EF ≥ 50% | 0.408 | 0.248-0.671 | **<0.001** |
| **Wide QRS** |  |  |  |
| Study group | 1.125 | 0.547-2.317 | 0.748 |
| Age | 1.073 | 1.016-1.133 | **0.011** |
| Sex, female | 0.463 | 0.222-0.966 | **0.040** |
| DM | 0.491 | 0.226-1.063 | 0.071 |
| Baseline QRS width, ms | 1.015 | 0.998-1.033 | 0.077 |

CI=confidence interval; DM=diabetes mellitus; EP=ejection fraction; HR=hazard ratio; PPI=permanent pacemaker implantation.
